# Retrotransposon-Based Molecular Markers for Analysis of Genetic Diversity within the Genus *Linum*

**DOI:** 10.1155/2014/231589

**Published:** 2014-08-27

**Authors:** Nataliya V. Melnikova, Anna V. Kudryavtseva, Alexander V. Zelenin, Valentina A. Lakunina, Olga Yu. Yurkevich, Anna S. Speranskaya, Alexey A. Dmitriev, Anastasia A. Krinitsina, Maxim S. Belenikin, Leonid A. Uroshlev, Anastasiya V. Snezhkina, Asiya F. Sadritdinova, Nadezda V. Koroban, Alexandra V. Amosova, Tatiana E. Samatadze, Elena V. Guzenko, Valentina A. Lemesh, Anastasya M. Savilova, Olga A. Rachinskaia, Natalya V. Kishlyan, Tatiana A. Rozhmina, Nadezhda L. Bolsheva, Olga V. Muravenko

**Affiliations:** ^1^Engelhardt Institute of Molecular Biology, Russian Academy of Sciences, Moscow 119991, Russia; ^2^Department of Higher Plants, Lomonosov Moscow State University, Moscow 119991, Russia; ^3^Research Institute of Physico-Chemical Medicine, Moscow 119435, Russia; ^4^Institute of Genetics and Cytology, National Academy of Science of Belarus, 220072 Minsk, Belarus; ^5^Research Center for Obstetrics, Gynecology and Perinatology, Moscow 117997, Russia; ^6^All-Russian Research Institute for Flax of the Russian Academy of Agricultural Sciences, Torzhok 172002, Russia

## Abstract

SSAP method was used to study the genetic diversity of 22 *Linum* species from sections *Linum*, *Adenolinum, Dasylinum, Stellerolinum*, and 46 flax cultivars. All the studied flax varieties were distinguished using SSAP for retrotransposons *FL9* and *FL11*. Thus, the validity of SSAP method was demonstrated for flax marking, identification of accessions in genebank collections, and control during propagation of flax varieties. Polymorphism of *Fl1a, Fl1b*, and *Cassandra* insertions were very low in flax varieties, but these retrotransposons were successfully used for the investigation of *Linum* species. Species clusterization based on SSAP markers was in concordance with their taxonomic division into sections *Dasylinum, Stellerolinum, Adenolinum*, and *Linum*. All species of sect. *Adenolinum* clustered apart from species of sect. *Linum*. The data confirmed the accuracy of the separation in these sections. Members of section *Linum* are not as closely related as members of other sections, so taxonomic revision of this section is desirable. *L. usitatissimum* accessions genetically distant from modern flax cultivars were revealed in our work. These accessions are of utmost interest for flax breeding and introduction of new useful traits into flax cultivars. The chromosome localization of *Cassandra* retrotransposon in *Linum* species was determined.

## 1. Introduction

The genus* Linum* comprises about 200 species which are distributed throughout the temperate and subtropical regions of the world. The genus is subdivided by Ockendon and Walters into five sections:* Linum, Dasylinum* (Planch.) Juz.,* Linastrum* (Planchon), Bentham,* Syllinum* Griseb., and* Cathartolinum* (Reichenb.) Griseb. [1]. Some taxonomists classified the members of the* L. perenne* group from section* Linum* to an independent section* Adenolinum* (Reichenb.) Juz. [2, 3]. The species* L. stelleroides* (Planch.), distributed in Far East and China, was classified by Yuzepchuk [2] to a monotype section* Stellerolinum* Juz. ex Prob. The phylogenetic analyses based on chloroplast (*ndhF*,* trnL-F*, and* trnK* 3′ intron) and nuclear ITS (internal transcribed spacer) DNA sequences revealed that genus* Linum* was not monophyletic. It contains two major lineages: a yellow-flowered clade (sections* Linopsis*,* Syllinum*, and* Cathartolinum*) and a blue-flowered clade (sections* Linum*,* Dasylinum*, and* Stellerolinum*) [4]. The cultivated flax (*L. usitatissimum* L.) belongs to sec.* Linum* from a blue-flowered clade.* L. usitatissimum* is believed to have originated as a result of domestication of wild species* L. angustifolium* Huds. approximately 8000 years ago [5–8]. For a long time flax has been cultivating as a dual-purpose crop grown for its fiber and linseed oil.

According to morphological and qualitative traits, cultivated flax was divided into five main types: (1) fiber flax (*L. usitatissimum* subsp.* usitatissimum*); (2) oil flax (*L. usitatissimum* L. subsp.* humile* Czernom.); (3) dual-purpose flax (*L. usitatissimum* L. subsp.* intermedium* Czemom.) that was an intermediate form between the first two ones cultivated for fiber and oil; (4) large seeded flax (*L. usitatissimum* L. subsp.* latifolium* Snankev.) which is characterized by a set of specific morphological features and cultivated for oil in the Mediterranean region and North Africa; (5) winter flax (*L. usitatissimum* L. subsp.* bienne* Mill. Snankev.) cultivated for fiber and oil in the Caucasus, Turkey, Balkans, and some other south regions of Europe [9, 10]. In addition, collections of flax germplasm maintain accessions of primitive flax forms with dehiscent capsules (*L. usitatissimum* convar.* crepitans* [Boenningh.] Kulpa et Danert) [11].

The taxonomy of the genus cannot be considered as finally established one because the phylogenetic linkages between the individual taxa have not been sufficiently investigated. The phylogeny of species of the genus* Linum* was previously studied by the use of molecular and cytogenetic approaches [4, 12–17], but there are problems that still remain to be solved.

Transposon-based molecular markers are successfully used in phylogenic studies. Transposable elements were shown to influence changing in genomic structure as well as transcriptional regulation occurring during the evolution [18, 19]. The presence of transposons in various species of plants, their high integration activity, conservative sequences, and a large number of copies encouraged the use of transposons in the studies of genetic diversity and profiling of plant varieties [20–22]. Several molecular marker systems based on the information available for the transposable elements sequences were developed for plants [20, 22–27]. SSAP (sequence-specific amplified polymorphism) method was shown to have a number of advantages as compared to other marker systems. SSAP method produces many polymorphic fragments and allows differentiation of most samples using only a single combination of specific primers [23, 28–30]. Different plants were successfully studied by SSAP analysis, but the method has not been applied for the investigation of species of the genus* Linum* yet. Only recently flax sequences have appeared in databases [31–34], and development of a marker system based on flax transposable elements for the investigation of cultivated and wild species of the genus* Linum* has become possible.

In this study the SSAP method was used for assessment of genetic diversity. Besides, the possibilities of application of marker-based profiling for identification of* L*.* usitatissimum* varieties were analyzed. We studied 46 varieties of* L. usitatissimum* mainly bred in Russia and a number of varieties which were grown in geographically close or distant regions. We also analyzed different types of cultivated flax (fiber, oilseed, large seeded, winter, and dehiscent flax) together with 21 wild species and subspecies from sections* Linum*,* Adenolinum*,* Dasylinum*, and* Stellerolinum* to estimate the possibility of using the SSAP method for the investigation of flax domestication history and phylogenic linkages between different taxa of the genus* Linum*.

## 2. Materials and Methods

### 2.1. Plant Materials

For the investigation of genetic diversity of cultivated flax, 46* L. usitatissimum* varieties, mainly of Russian origin, were obtained from the All-Russian Research Institute for Flax (VNIIL) (Table 1). For analyzing the reproducibility of the SSAP analysis, flax variety “Stormont cirrus” (IPK genbank, Gatersleben, accession number: LIN 261) was used.

For the investigation of genetic diversity, 47 flax accessions belonging to 22 species from sections* Linum*,* Adenolinum*,* Dasylinum,* and* Stellerolinum* were used (Table 2). Most of these accessions were obtained from genebank of Leibniz Institute of Plant Genetics and Crop Plant Research (IPK) (Gatersleben, Germany), seed collections of All-Russian Flax Institute (VNIIL) (Torzok, Russian Federation), N. I. Vavilov Research Institute of Plant Industry (VIR) (St. Petersburg, Russian Federation), and Institute of Genetics and Cytology of NAS Belarus (IGC) (Minsk, Belarus). The accessions of* L. amurense* and* L. stelleroides* were kindly provided by Dr. L. N. Mironova, Botanic Garden Institute of the Far-Eastern Branch of the Russian Academy of Sciences (BGI) (Vladivostok, Russian Federation). Some accessions were collected in the wild by Dr. A.A. Svetlova, Komarov Botanical Institute RAS (St. Petersburg, Russian Federation), by Dr. N. L. Bolsheva, Engelhardt Institute of Molecular Biology RAS (Moscow, Russian Federation), and by Dr. M. Pavelka, Euroseeds (Novy Jicin, Chech Rep.).

### 2.2. Confirmation of Species Determination

Species determination of some accessions of wild* Linum* species was done during the course of our earlier cytogenetic investigations [14, 17, 37]. To confirm the species determination, the rest of the accessions were planted in the ground, and, additionally, chromosome analysis (determination of chromosome number) using acetocarmine staining according to previously developed approach was performed [14]. Chromosome numbers of all the studied accessions of wild flax are represented in Table 2.

### 2.3. SSAP Analysis

Genomic polymorphism of different* Linum* species was studied using SSAP method [23] with modifications described earlier through genomic studies of wheat [38] and strawberry [39]. Total genomic DNA was extracted from young flax leaves according to Edwards et al. [40] with minor modifications. 30 ng of genomic DNA was treated with 10 U of* TaqI* restriction enzyme (Thermo Scientific, USA) for 3 h at 65°C. Ligation was performed by adding 2.5 U of T4 DNA-ligase (Thermo Scientific, USA), 5 mM ATP, and 50 pmol of double-strand adapter [5′-ACTCGATTCTCAACCCGAAAGTATAGATCCCA; 5′-PO_4_-CGTGGGATCTATACTT-(C6linker)-NH_2_] and incubation for 7 h at 37°C. The product was diluted twice with deionized water. The DNA sequence between the LTR (long terminal repeat) region of retrotransposons and the *Taq* ^*α*^ *I* restriction site was amplified using the adapter primer (5′-GTTTACTCGATTCTCAACCCGAAAG 3′) and primers to the LTR regions of* FL1a*,* FL1b*,* FL4*,* Cassandra*,* FL10*,* FL8*,* FL7*,* FL12*, and* FL9* retrotransposons [32]:

 1826 5′-ACCCCTTGAGCTAACTTTTGGGGTAAG-3′ (*FL1a, FL1b*)
 1833 5′-CTTGCTGGAAAGTGTGTGAGAGG-3′ (*FL4*)
 1838 5′-TGTTAATCGCGCTCGGGTGGGAGCA-3′ (*FL1a, FL1b, Cassandra*)
 1845 5′-AGCCTGAAAGTGTTGGGTTGTCG-3′ (*FL11*)
 1846 5′-CTGGCATTTCCATTGTCGTCGATGC-3′ (FL10)
 1854 5′-GCATCAGCCTGGACCAGTCCTCGTCC-3′ (*FL8*)
 1868 5′-CACTTCAAATTTTGGCAGCAGCGGATC-3′ (*FL1a, FL1b*)
 1881 5′-TCGAGGTACACCTCGACTCAGG-3′ (*FL7*)
 1886 5′-ATTCTCGTCCGCTGCGCCCCTACA-3′ (*FL12*)
 1899 5′-TGAGTTGCAGGTCCAGGCATCA-3′ (*FL9*)

Amplification was performed in two stages. At the first stage, only the primer to LTR of the retrotransposon was used. Amplification was carried out in 25 *μ*L of PCR mix containing 5 *μ*L of ligation mix, 1 U of TrueStart Hot Start* Taq* DNA polymerase (Thermo Scientific, USA), TrueStart* Taq* DNA polymerase buffer, 0.5 mM MgCl_2_, 20 *μ*M dNTP (Thermo Scientific), and 5 pmol of the LTR primer. The program for amplification for the first stage was 95°C for 15 min, 30 cycles (95°C for 30 s, 62°C for 1 min, 72°C for 2 min) and 72°C for 10 min. At the second stage of amplification, the adapter primer 5′-GTTTACTCGATTCTCAACCCGA-3′ and one of the primers to the LTR region of retrotransposons were used. Amplification was carried out in 25 *μ*L of PCR mix containing 12 *μ*L of the first-stage PCR product, 1 U of* Taq* DNA polymerase (Thermo Scientific, USA),* Taq* DNA polymerase buffer, 1.5 mM MgCl_2_, 200 *μ*M dNTP (Thermo Scientific, USA), 25 pmol of LTR primer, and 25 pmol of the adapter primer. The program for amplification for the second stage was 95°C for 15 min, 35 cycles (95°C for 30 s, 62°C for 1 min, 72°C for 2 min), and 72°C for 10 min. The PCR products were separated in 2.5% agarose gel using TВЕ buffer and then stained with ethidium bromide. Ten PCR products were excised from agarose gel and characterized by sequencing on Applied Biosystems 3730 DNA Analyzer to confirm the specificity of SSAP reaction. The Bio-Rad Gel Doc system was used for gel documentation and photography as well as for visual detection of presence or absence of polymorphic fragments in the samples from different accessions. These data were recorded in the form of a binary matrix in which the presence of a fragment was coded as 1 and its absence as 0.

The genetic distances between varieties were calculated based on the binary matrix of amplified fragments using Dice's formula [41]. The dendrograms were constructed using SplitsTree 4.10 software [42]. Cluster analysis was performed using neighbor-joining method [43] and bootstrap values were determined based on 5000 permutations.

### 2.4. Preparation of* Cassandra* Retrotransposon DNA Probe

PCR primers were designed for amplification of* Cassandra* retrotransposon. We amplified an internal domain (primers IntDom-F AGTGGTATCCGAGCCTCT and IntDom-R CCCATAGGACTCAACGTC) and the LTR with the exception of the 5S rDNA region of this retrotransposon (primers LTR-1-86-F TGTAATGTAACACGTTAGGCA and LTR-1-86-R TTAGTTAGGGACGGATTGTT; LTR-206-279-F AAATAAATCTGTGAGGGATTAGT and LTR-206-279-R ACTTGTAACACCCCGTACT). The amplification was carried out in 20 *μ*L of PCR mixture that contained 1 U of* TaqF* DNA polymerase (Amplisens, Russia), 1x* TaqF* buffer, 25 pmol of the forward and reverse primer, 200 *μ*M dNTP (Amplisens, Russia), and 10 ng of genomic DNA. The program for amplification was 95°C for 15 min, 40 cycles (95°C for 10 s, 62°C for 20 s, 72°C for 30 s), and 72°C for 10 min. The amplicons were analyzed in 2% agarose gel and then used as a template for biotin PCR labeling to obtain biotin-labeled probes for FISH. PCR labeling was carried out using Biotin PCR Labeling Core Kit (Jena Bioscience, Germany) according to the manufacture's protocol. Labeled PCR products were precipitated with ethanol.

### 2.5. FISH with* Cassandra* Retrotransposon DNA Probe

Chromosome preparation was carried out according to the technique developed earlier for plants having small-sized chromosomes [14]. The hybridization mixture contained 2x SSC, 50% formamide, 10% dextran sulphate, and 2 ng/*μ*L of a biotinylated DNA probe of* Cassandra* retrotransposon. The probe was hybridized overnight at 31°C. After hybridization the slides were washed twice with 0.1x SSC at 38°C for 10 min, followed by two washes with 2x SSC at 44°C for 5 min and a final 5 min wash in 2x SSC at room temperature. The biotin-labeled DNA probe was detected using a highly sensitive Alexa Fluor 488, Tyramide Signal Amplification system (Invitrogen) according to manufacturer's instructions.

## 3. Results

### 3.1. Analysis of SSAP Fingerprints

**Figure 1:**
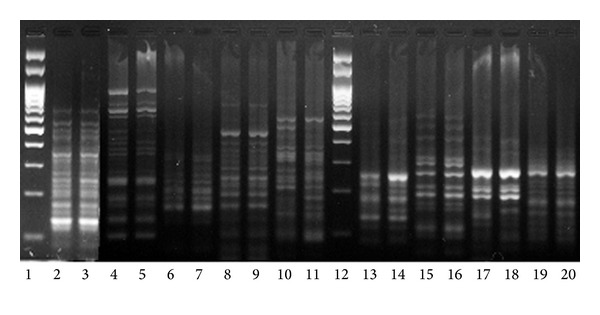
The test of reproducibility of SSAP markers obtained for “Stormont cirrus” flax variety. Lanes 2 and 3, primer 1899; lanes 4 and 5, primer 1826; lanes 6 and 7, primer 1838; lanes 8 and 9, primer 1845; lanes 10 and 11, primer 1846; lanes 13 and 14, primer 1854; lanes 15 and 16, primer 1868; lanes 17 and 18, primer 1881; lanes 19 and 20, primer 1886; lanes 1 and 12, 100 bp DNA ladder.

For analyzing of the reproducibility of the SSAP method, DNA of flax variety “Stormont cirrus” was restricted twice, ligated, and amplified with the primers. The obtained PCR products were visualized in acrylamide and agarose gels (Figure 1). Electrophoretic spectra of PCR products obtained with primers 1826, 1838, 1845, 1868, 1886, and 1899 coincided completely demonstrating high reproducibility of the SSAP results. The fingerprints obtained with primers 1846, 1854, and 1881 varied in individual amplified fragments, so the use of primers 1846, 1854, and 1881 for SSAP analysis of flax varieties will require further optimization of restriction, ligation, or PCR conditions. We selected primers with high reproducibility (1838, 1845, 1868, and 1899) as they yielded PCR products that were easily discernible in an agarose gel. These primers were used for analysis of 46 flax varieties by the SSAP method.

All the examined varieties produced identical or very similar fingerprints with primers 1838 and 1868 (*FL1a*,* FL1b*, and* Cassandra*). At the same time, the PCR products obtained with primers 1845 and 1899 were unique for different flax varieties. So, primers 1845 and 1899 were chosen for analyzing of genetic diversity of flax varieties. Several of PCR products have been sequenced (Supplementary Material available online at http://dx.doi.org/10.1155/2014/231589), the majority of obtained sequences are significantly similar to the sequences of corresponding retrotransposons.

### 3.2. Analysis of Genetic Diversity of Flax Varieties

**Figure 2:**
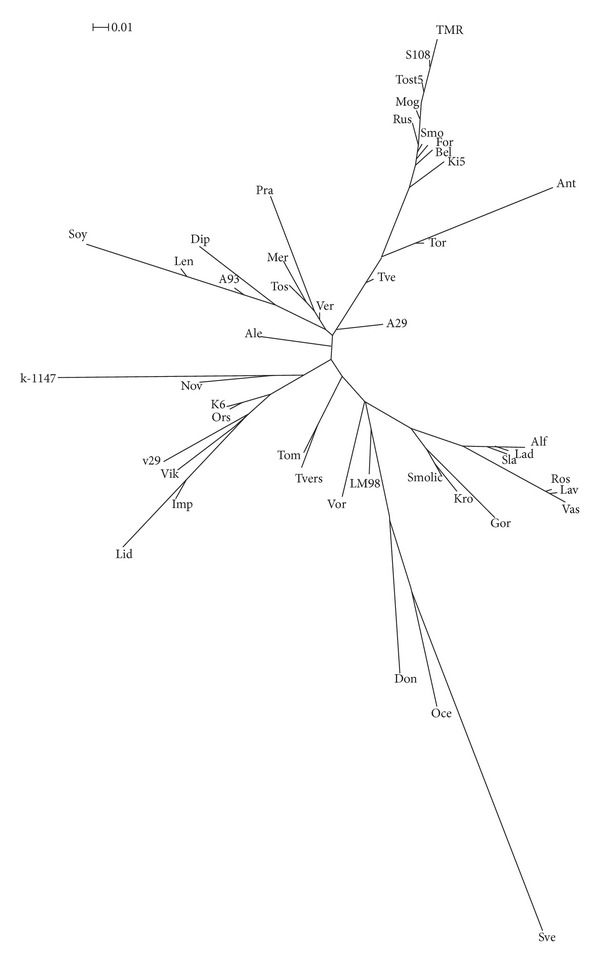
A neighbor-joining dendrogram for SSAP markers. Flax varieties: Alf-Alfa; Gor: Gorizont; Lav: Lavina; Vas: Vasilyok; Soy: Soyuz; Lad: Lada; Sla: Slavnyj 82; Kro: Krom; Tvers: Tverskoj; Ros: Rosinka; Len: Lenok; A93: A-93; Tos: Tost 3; Mog: Mogilevskij 2; Bel: Belochka; Tor: Torzhokskij 4; Tve: Tvertsa; Smolic: Smolich; A29: A-29; Ant: Antey; Rus: Rusich; Ver: Veralin; Mer: Merilin; Smo: Smolenskij; Tost5: Tost 5; S108: S 108; Imp: Impuls; Pra: Praleska; Lid: Lider; Vor: Voronezhskij; Sve: Svetoch; Ki5: Ki-5; Lm98: LM-98; Nov: Novotorzhskij; TMR: TMR-1919; k-1147: k-1147; v29: v-29; Ale: Aleksim; Oce: Ocean k-4497; Ors: Orshanskij 2; K6: K-6; Vic: Viksoil V-2; Don: Donskoj 95; Dip: Diplomat; For: Ford; Tom: Tomskij 16.

Visual analysis of SSAP fingerprints based on retrotransposons* FL11 *and* FL9* revealed 44 polymorphic retrotransposon insertions (23 fragments for primer 1845 and 21 fragments for primer 1899) in 46 flax varieties. Each of the 46 varieties had their own unique spectrum of retrotransposon insertions. So, we could differentiate all the 46 varieties using only two SSAP primers. In order to analyze genetic diversity of these varieties, we compiled a binary matrix of the presence/absence of polymorphic insertions of the above-mentioned retrotransposons, calculated the genetic distances between the varieties using Dice's formula [41], and constructed a dendrogram by using the neighbor-joining method (Figure 2). The obtained tree branching pattern revealed no distinct clusters among examined varieties.

### 3.3. Genomic Diversity of Species of the Genus* Linum*

**Figure 3:**
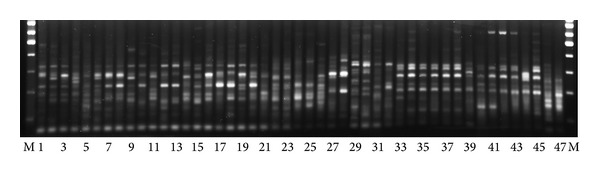
SSAP markers generated using 1868 primer for Linum species: 1:* L. perenne* LIN 1807; 2:* L. perenne *subsp.* extraaxilare*, LIN 1651; 3:* L. altaicum*, LIN 1632; 4:* L. komarovii* LIN 1716; 5:* L. perenne*, LIN 1521; 6:* L. perenne* susp.* alpinum*, LIN 1905; 7:* L. perenne* susp.* anglicum*, LIN 1524; 8:* L. perenne*, K 5500; 9:* L. leonii*, LIN 1672; 10:* L. pallescens*, LIN 1645; 11:* L. pallescens*, Altai; 12:* L. mesostylum*, LIN 1774; 13:* L. mesostylum*, LIN 1662; 14:* L. lewisii*, LIN 1648; 15:* L. lewisii*, LIN 1550; 16:* L. austriacum*, LIN 1608; 17:* L. austriacum*, Rostov; 18:* L. austriacum* subsp.* euxinum*, LIN 1546; 19:* L. austriacum*, Crimea; 20:* L. austriacum*, Ukraine; 21:* L. amurense*; 22:* L. hirsutum*, LIN 1676; 23:* L. hirsutum*, LIN1649; 24:* L. hirsutum* subsp.* pseudoanatolicum*; 25:* L. hirsutum *subsp.* anatolicum*; 26:* L. marginale*; 27:* L. narbonense*, LIN 2002; 28:* L. narbonense*, LIN1653; 29:* L. decumbens*, LIN 1754; 30:* L. decumbens*, LIN1913; 31:* L. grandiflorum*, LIN 2000; 32:* L. grandiflorum*, LIN 974; 33:* L. angustifolium*, LIN 1692; 34:* L. angustifolium*, K 5695; 35:* L. angustifolium*, K 3108; 36:* L. angustifolium,* Belarus; 37:* L. angustifolium*, K 4731; 38:* L. biene* (syn.* L. angustifolium*); 39: winter flax (*L. usitatissimum *subsp.* biene*); 40: dehiscent flax (*L. usitatissimum *convar.* crepitans*); 260; 41: dehiscent flax (*L. usitatissimum *convar.* crepitans*), LIN 119; 42: large seeded flax(*L. usitatissimum*), LIN 277; 43: dual-purpose flax (*L. usitatissimum*), LIN 633; 44: winter flax (*L. usitatissimum*), u 099845; 45: large seeded flax (*L. usitatissimum*), *к* 7131; 46:* L. stelleroides*, Telyakovsky Inlet; 47:* L. stelleroides*, Kraskino settlement. M—100 bp DNA ladder.

For investigation of species of the genus* Linum*, we chose retrotransposons* FL1a*,* FL1b*, and* Cassandra *which did not show high insertion polymorphism within cultivated flax varieties. Primers 1838 and 1868 were used for SSAP analysis. As the result, 95 bands that originated with primer 1838 and 128 bands with primer 1868 (Figure 3) were scored. All the bands were polymorphic. Based on the SSAP fingerprint similarity, nine groups of closely related species (A-I) were distinguished. Group A included different species of sect.* Adenolinum* (syn.* L. perenne* group); group B consisted of* L. hirsutum* subsp.* hirsutum* accessions; group C′′ included* L. hirsutum *subsp.* pseudoanatolicum* and* L. hirsutum* subsp.* anatolicum*. Groups D, E, F, and G comprised species accessions of sect.* Linum *(*L. marginale; L. narbonense, L. decumbens, and L. grandiflorum*, resp.). Group H included accessions of* L. angustifolium* and* L. usitatissimum* (sect.* Linum*); and group I consisted of* L. stelleroides* accessions (sect.* Stellerolinum*).

**Figure 4:**
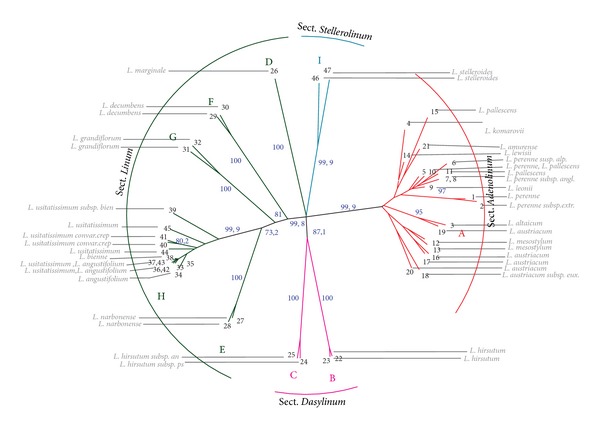
Neighbor-joining dendrogram for SSAP markers. Flax species: 1:* L. perenne* LIN 1807; 2:* L. perenne *subsp.* extraaxilare*, LIN 1651; 3:* L. altaicum*, LIN 1632; 4:* L. komarovii* LIN 1716; 5:* L. perenne*, LIN 1521; 6:* L. perenne* susp.* alpinum*, LIN 1905; 7:* L. perenne* susp.* anglicum*, LIN 1524; 8:* L. perenne*, K 5500; 9:* L. leonii*, LIN 1672; 10:* L. pallescens*, LIN 1645; 11:* L. pallescens*, Altai; 12:* L*.* mesostylum*, LIN 1774; 13:* L. mesostylum*, LIN 1662; 14:* L. lewisii*, LIN 1648; 15:* L. lewisii*, LIN 1550; 16:* L. austriacum*, LIN 1608; 17:* L. austriacum*, Rostov; 18:* L. austriacum *subsp.* euxinum*, LIN 1546; 19:* L. austriacum*, Crimea; 20:* L. austriacum*, Ukraine; 21:* L. amurense*; 22:* L*.* hirsutum*, LIN 1676; 23:* L. hirsutum*, LIN1649; 24:* L. hirsutum* subsp.* pseudoanatolicum*; 25:* L. hirsutum *subsp.* anatolicum*; 26:* L. marginale*; 27:* L. narbonense*, LIN 2002; 28:* L. narbonense*, LIN1653; 29:* L. decumbens*, LIN 1754; 30:* L. decumbens*, LIN1913; 31:* L. grandiflorum*, LIN 2000; 32:* L. grandiflorum*, LIN 974; 33:* L. angustifolium*, LIN 1692; 34:* L. angustifolium*, K 5695; 35:* L*.* angustifolium*, K 3108; 36:* L. angustifolium,* Belarus; 37:* L. angustifolium*, K 4731; 38:* L. biene* (syn.* L. angustifolium*); 39: winter flax (*L. usitatissimum *subsp.* biene*); 40: dehiscent flax (*L. usitatissimum *convar.* crepitans*); 260; 41: dehiscent flax (*L. usitatissimum *convar.* crepitans*), LIN 119; 42: large seeded flax (*L usitatissimum*), LIN 277; 43: dual-purpose flax (*L usitatissimum*), LIN 633; 44: winter flax (*L usitatissimum*), u 099845; 45: large seeded flax (*L usitatissimum*), *к* 7131; 46:* L. stelleroides*, Telyakovsky Inlet; 47:* L. stelleroides*, Kraskino settlement. Bootstrap values that exceeded 70% are shown in italic.

All the groups contained at least one group-specific marker. The results of phylogenetic analysis of* Linum* species are shown in the dendrogram on Figure 4. As the dendrogram shows nine clearly distinguished groups of species supported by high bootstrap values can be observed.

### 3.4. FISH with* Cassandra* Retrotransposon DNA Probe

**Figure 5:**
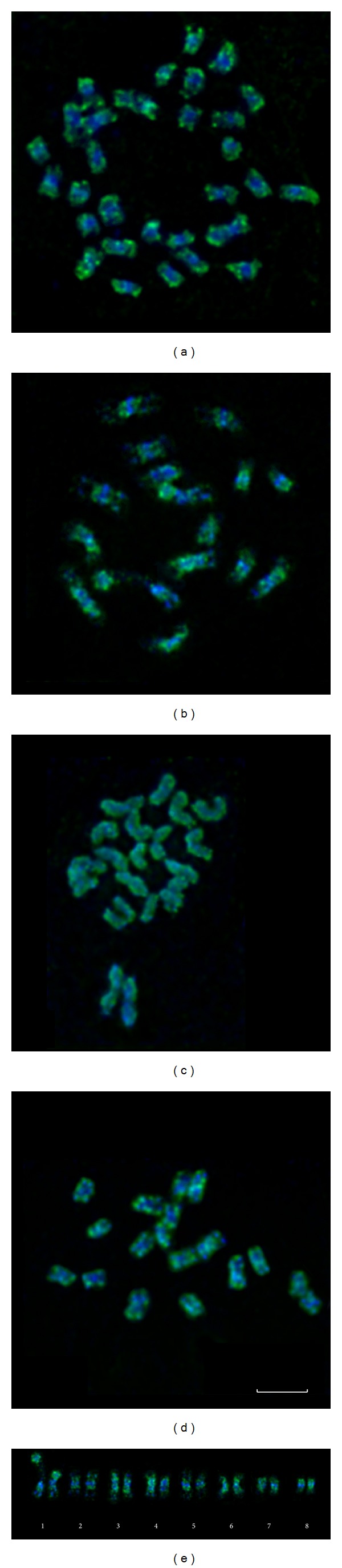
FISH with DNA probe of Cassandra retrotransposons (green). (a)* L. usitatissimum* (sect.* Linum*); (b)* L. grandiflorum* (sect.* Linum*); (c)* L. narbonense* (sect.* Linum*); (d)* L. amurense* (sect.* Adenolinum*); (e) a karyotype of* L. grandiflorum. *Chromosomes were stained with DAPI (blue). Bar—5 *μ*m.

The highly sensitive tyramide FISH method was applied for the investigation of abundanceof* Cassandra* retrotransposons as well as their distribution along chromosomes in three species of sect.* Linum *(*L. usitatissimum*,* L. grandiflorum, *and* L. narbonense*) and* L. amurense* (sect.* Adenolinum*). FISH revealed that* Cassandra* dispersed along the whole length of chromosomes in karyotypes of four studied species, but its distribution along the chromosomes was nonrandom (Figure 5). In species having small-sized chromosomes (*L. usitatissimum*,* L. grandiflorum,* and* L. amurense*),* Cassandra* was mainly localized in pericentromeric and subtelomeric chromosome regions. The patterns of* Cassandra* distribution were chromosome specific and were similar in homologous pairs of chromosomes (Figure 5(e)). In* L. narbonense*, which possessed large chromosomes, the patterns of* Cassandra* distribution resembled the patterns observed in karyotypes of the above-mentioned species (having small-sized chromosomes) though they were more regular.

## 4. Discussion

### 4.1. Use of SSAP Analysis for Identification of Flax Varieties and Estimation of Genetic Diversity

To estimate genetic polymorphism and to characterize* L. usitatissimum* varieties, the SSAP method was used, and also high reproducibility of the method was shown. The validity of the SSAP method for molecular genetic studies of flax varieties using* FL11 *and* FL9 *retrotransposons was demonstrated. Obtained with primers 1838 and 1868 (retrotransposons* FL1a*,* FL1b,* and* Cassandra*) PCR products were very similar in all the studied varieties. Such low diversity of cultivated flax might be a result of low transposition activity and/or creation of bottleneck effect during flax selection.

In analyzed flax varieties, 44 polymorphic insertions for* FL11* (primer 1845) and* FL9* (primer 1899) retrotransposons were revealed. Every studied variety possessed a unique set of SSAP markers. Therefore, the SSAP method can be used to mark the genotypes, to identify varieties of* L. usitatissimum* in genebank collections, to exercise control during of flax variety growth, and to obtain high quality seed material, when the varietal identity is particularly important.

The genetic similarity of 46 flax varieties was characterized by genetic distances calculated based on the SSAP data. The dendrogram (Figure 3) did not contain clearly isolated clusters of varieties. Thus, the studied flax varieties could not be subdivided into distinct groups. Our results were in agreement with earlier obtained data shown that flax accessions examined by IRAP analysis did not form distinct clusters in studies of their origin or the type of commercial use (fiber or oil). These data indicated an overlap in genetic diversity despite of disruptive selection for fiber or seed oil types [32]. In our study, the SSAP method also failed to distinguish fiber or oil seed flax varieties. Since varieties with the best characteristics are commonly used as parents in breeding practice, some valuable flax forms present in the genealogy of most modern varieties. Besides, the lines selected for crossing are usually characterized by low genetic diversity. So, the commercial flax varieties were shown to be less diverse than wild flax species and landraces [32].

Although the examined flax varieties could not be clustered into different groups by SSAP method, it might be used for estimation of their genetic similarity based on polymorphic insertions of retrotransposons. The estimation can be used for choosing the parents in breeding practice and also for creation of core collections which should include genetically diverse accessions.

### 4.2. Diversity and Phylogeny of* Linum* Species

In the present study, 20 accessions from sect.* Linum*, 21 accessions from sect.* Adenolinum*, 4 accessions from sect.* Dasylinum*, and 2 accessions from sect.* Stellerolinum* were analyzed by using SSAP method. All the examined species were clustered into 9 groups mainly according to common taxonomic division of the genus* Linum* into sections (Figure 4).

### 4.3. Section* Dasylinum*

Species from sect.* Dasylinum* clustered together and formed two related groups B and C. Group B included* L. hirsutum* subsp.* pseudoanatolicum* and* L. hirsutum* subsp.* anatolicum* and group C included* L. hirsutum* subsp.* hirsutum. *Thus, SSAP analysis singled out sect.* Dasylinum* as a well-supported clade. Our results were in agreement with the AFLP and ITS data as well as chloroplast phylogenies, chromosome studies, and transcriptome analysis of* Linum* species [4, 13, 37, 44]. It should be mentioned that the subdivision of accessions of sect.* Dasylinum* into two related clusters correlated with their difference in chromosome numbers and the origin of accessions. Thus, the accessions of* L. hirsutum* subsp.* hirsutum* (cluster C) from Europe was characterized by chromosome number of 2n = 16, while accessions from Turkey,* L. hirsutum* subsp.* pseudoanatolicum* and* L. hirsutum* subsp.* anatolicum* (cluster B) have chromosome number 2n = 32. Chromosome numbers for* L. hirsutum* subsp.* pseudoanatolicum* and* L. hirsutum* subsp.* anatolicum* were firstly determined in the present study.

### 4.4. Section* Stellerolinum*

Two accessions of* L. stelleroides* have rather similar SSAP fingerprints which were differed significantly from all the others* Linum* species and formed a separated clade. The similar results were obtained by phylogenetic analyses of chloroplast and ITS DNA sequences [4]. Moreover,* L. stelleroides* was shown to have chromosome number 2n = 20 which was unique for blue-flowered flaxes [45].

### 4.5. Section* Adenolinum*

All the members of sect.* Adenolinum* formed an independent group clustered separately from species of sect.* Linum* and other sections. Distinct isolation of this species group was also revealed in several molecular and karyological investigations [4, 12–14, 17]. The data were in good agreement with the opinion of Yuzepchuk [2] and Egorova [3] who isolated the group from sect.* Linum* into an independent section* Adenolinum*.

SSAP fingerprints of the accessions within sect.* Adenolinum* were highly polymorphic, but SSAP markers did not allow us to reveal any species subclusters supported by a high bootstrap value. Thus, SSAP analysis used in the present study as well as AFLP and RAPD analyses [13, 17] separated individual accessions but did not identify individual species inside sect.* Adenolinum*.

### 4.6. Section* Linum*

Sect.* Linum* was subdivided into 5 groups by neighbor-joining clustering. Accessions of* L. marginale*,* L. grandiflorum*,* L. decumben*, and* L. narbonense* formed four independent single species groups, while the fifth group combined accessions* L. angustifolium* and* L. usitatissimum*. Similar results had been obtained earlier by AFLP, RAPD, molecular phylogeny based on chloroplast* RbsL* sequence, and molecular cytogenetic methods (C/DAPI-banding patterns and localization of rRNA genes on chromosomes) [4, 12–14].

Within a subgroup consisted of* L. usitatissimum* and* L. angustifolium*, the accessions of large seeded flax (breeding cultivar), dual-purpose flax, and* L. angustifolium* were rather similar. Their fingerprints did not differ significantly from fingerprints of studied 46 flax varieties. This data were in a good agreement with the suggestion that* L. angustifolium* was the progenitor of* L. usitatissimum* [5, 7].

The accession of large seeded flax landrace, the accessions of winter flax, and the accessions of dehiscent flax differed significantly from the other members of cluster H. Both accessions of dehiscent flax grouped together (supported by a high bootstrap value) and had species-specific SSAP markers.

It should be noted that flax accessions, which are genetically distant from modern flax cultivars, are particular important for flax breeding. The genetic diversity of cultivated flax decreased significantly during the last decades. It might lead to the lack of useful alleles in genomes of modern cultivars [13]. Therefore, introduction of new useful traits from the ancient primitive forms of cultivated flax and wild species could increase the polymorphism of modern flax varieties. SSAP markers allowed us to identify the unique accessions which are important for the investigation of the history of flax domestication.

*L. marginale*, the last member of sect.* Linum*, is a wild flax native to Australia. We found that it had the maximal chromosome number (2n = 84) in the genus* Linum*. The number indicated a high level of ploidy of* L. marginale* genome. SSAP patterns of the species were significantly different compared with the other species of sect.* Linum*. Therefore,* L. marginale* clustered apart from the other species. The obtained results were in contradiction with ITS and chloroplast topologies which clustered the species together with* L. bienne* and* L. usitatissimum* [4]. Rogers [46] assumed that Australian and New Zealand species* L. marginale* Cunn. and* L. monogynum* Forst. were related to European species* L. hologynum* Reichenb. (sec.* Linum*). This assumption based on the fact that diploid chromosome number of* L. hologynum *(2n = 42) corresponded to haploid chromosome number of* L. monogynum* and* L. marginale*. Moreover, all the three species had fused styles and pantoporate pollen grains that were unusual for blue-flowered flaxes. Thus, all the above-mentioned data indicated that the phylogenic lineages of* L. marginale* need further investigation.

The data obtained in the present study, as well as the results of other molecular phylogenetic and chromosomal investigations, indicated that members of section* Linum* were not as closely related as members of other sections. Therefore, taxonomic revision of this section is desirable.

### 4.7. Chromosome Location of* Cassandra* Retrotransposon

As differences in SSAP fingerprints for several flax species were found, we decided to analyze the distribution of* Cassandra* retrotransposon along the chromosomes of* Linum* species.* Cassandra* is a terminal-repeat retrotransposon in miniature (TRIM) that carries conserved 5S rDNA sequences in its LTRs.* Cassandra* was found in a number of vascular plants [31]. In our work, we investigated the distribution of this retrotransposon along the chromosomes of* Linum* species using tyramide FISH. We revealed that* Cassandra* localized in pericentromeric and subtelomeric regions of chromosomes that was typical for transposable elements [47]. A more uniform distribution of* Cassandra* retrotransposon was found in* L. narbonense* in comparison with* L. usitatissimum*,* L. grandiflorum*, and* L. amurense*. It was probably due to a higher content of transposable elements correlated with a larger size of its chromosomes (therefore its genome).

## 5. Conclusions

The availability of LTR sequences of flax retrotransposons and high polymorphism of SSAP markers offer a promising potential for SSAP analysis of genus* Linum*. Applications of SSAP analysis, for example, evolutionary and phylogenetic studies, assessment of genetic diversity, accession identification, and search for exotic genepools of cultivated flax, could be applied to* L. usitatissimum* and other* Linum* species. SSAP analysis was shown to be very useful for characterization of flax varieties and identification of accession belonging to different species or sections and provided new information about of phylogenetic relationships within the genus* Linum*.

## Supplementary Material

Sequences of SSAP PCR products.

## Figures and Tables

**Table 1:** Studied flax varieties.

Number	Variety	Type	Originator	Variety pedigree
1	Alfa	Fiber	VNIIL	L-1120, Tomskij 4, T-10, Torzhokskij-4, G-4887
2	Gorizont	Fiber	VNIIL	L-1120, T-5, T-10, I-2, I-17, Tvertsa. Zarya, Marit Smolenskij, Severyanin, Silva, Natasha, Viera
3	Lavina	Fiber	Smolenskaja GOSHOS	Sadko × S-108 [X-5 (selected from L-1120) × 1-7]
4	Vasilyok	Fiber	Belarus	I-7, L-1120, X-5 (selected from L-1120), 221-84-4
5	Soyuz	Fiber	Smolenskaja GOSHOS	
6	Lada	Fiber	VNIIL	Viking, Fibra
7	Slavnyj 82	Fiber	VNIIL	from Shokinskij variety, by single plant selection
8	Krom	Fiber	Pskovskij NIISH	
9	Tverskoj	Fiber	VNIIL	L-1120, T-4, T-6, T-10, Torzhokskij-4, I-2, G-2782
10	Rosinka	Fiber	VNIIL	L-1120, T-5, I-17, Fibra, 1288/12
11	Lenok	Fiber	VNIIL	L-1120, M-34 (L-1120 × T-4), T-6, Lazurnyj
12	A-93	Fiber	VNIIL	L-1120, T-4, T-6, T-10, Torzhokskij-4
13	Tost 3	Fiber	Sibirskij NIISH	
14	Mogilevskij-2	Fiber	Belarus	Stahanovets, L-1120, T-5, T-9
15	Belochka	Fiber	Vjatskaja GSHA	L-1120, Tvertsa
16	Torzhokskij-4	Fiber	VNIIL	M-34 (L-1120 × T-4) × T-10 (G-360 × G-354)
17	Tvertsa	Fiber	VNIIL	T-5 × L-1120
18	Smolich	Fiber	Smolenskaja GOSHOS	Zarya (X-5 × T-5), mutant A-710, 806/3, severyanin
19	A-29	Fiber	VNIIL	L-1120, T-4, T-5, T-10
20	Antey	Fiber	Pskovskij NIISH	
21	Rusich	Fiber	Pskovskij NIISH	Pskovskij 83 (Pryadilshhik, L-1120, T-5, Pobeditel) × Rodnik (T-9, Progress, I-9, L-1120, VNIIL11, Spartak)
22	Veralin	Fiber	Netherlands	Torzhokskij-4 × Lidiya
23	Merilin	Fiber	Netherlands	
24	Smolenskij	Fiber	Smolenskaja GOSHOS	Tvertsa (T-5 × L-1120) × Zarya (X-5—selected from L-1120 × T-5)
25	Tost-5	Fiber	Sibirskij NIISH	
26	S-108	Fiber	Smolenskaja GOSHOS	X-5 (selected from L-1120) × I-7
27	Impuls	Fiber	Smolenskaja GOSHOS	S-108, X-5, I-7, Zarya
28	Praleska	Fiber	Belarus	
29	Lider	Fiber	Smolenskaja GOSHOS	Tayga (France selection), mutagen treatment, selection
30	Voronezhskij 1308/138	Oil	VNIIMK	
31	Svetoch	Fiber	VNIIL	Selected from Cherskij kryazh
32	Ki-5	Oil	Ukraine	
33	LM-98	Oil	VNIIL	
34	Novotorzhskij	Fiber	VNIIL	L-1120, I-2, I-17, selected from Bogotolskij kryazh, G-2307, G-4523-6-13
35	TMP 1919 china 1	Fiber	china	
36	k-1147, local form	Oil	Ethiopia	
37	v-29	Oil	China	
38	Aleksim	Fiber	VNIIL	L-1120, T-4, T-5, T-10, Tekstilshhik, M-34, Pryadilshhik, Tvertsa
39	Ocean k-4497	Oil	France	
40	Orshanskij-2	Fiber	Belarus	I-16 × L-1120
41	Κ-6	Fiber	Pskovskij NIISH	L 1120 × T-5
42	Vih oil v-2	Oil	France	
43	Donskoj-95	Oil	Donskaja op.st.	
44	Diplomat	Fiber	VNIIL	Viking × Fibra (with subsequent selection)
45	Ford	Fiber	Belarus	
46	Tomskij-16	Fiber	Sibirskij NIISH and T	T-9 × G-1077

**Table 2:** The accessions of studied species of genus* Linum*.

Number	Accession name	Source	Genebank number	Origin	Chromosome number (2n)	
Sect. *Adenolinum *						
1	*L. perenne *L.	IPK	LIN 1807	Russian Federation	2n = 18 [14]	
2	*L*. perenne L. subsp.* extraaxillare* (Kit.) Nyman	IPK	LIN 1651	Poland	2n = 36 [17]	
3	*L. altaicum* Ledeb. ex Juz.	IPK	LIN 1632	Unknown	2n = 18 [17]	
4	*L. komarovii* Juz.	IPK	LIN 1716	Unknown	2n = 18 [17]	
5	*L. perenne* L. subsp.* perenne *	IPK	LIN 1521	Slovakia	2n = 18 [17]	
6	*L*. perenne L. subsp.* alpinum* (Jacq.) Stoj. and Stef.	IPK	LIN 1905	Austria	2n = 18 [17]	
7	*L. perenne* L. subsp.* anglicum* (Mill.) Ockendon	IPK	LIN 1524	GB	2n = 36 [17]	
8	*L. perenne *L.	VNIIL	K 5500	Unknown	2n = 36 [17]	
9	*L. leonii *F. W. Schultz	IPK	LIN 1672	Germany	2n = 18 [14]	
10	*L. pallescens *Bunge	IPK	LIN 1645	Tajikistan	2n = 18 [17]	
11	*L. pallescens* Bunge	Wild population, collected by N. L. Bolsheva		Russian Federation, Altai	2n = 18 (present study)	
12	*L. mesostylum* Juz.	IPK	LIN 1774	Tajikistan	2n = 18 [17]	
13	*L. mesostylum *Juz.	IPK	LIN 1662	Tajikistan	2n = 18 [17]	
14	*L. lewisii *Pursh	IPK	LIN 1648	USA	2n = 18 [17]	
15	*L. lewisii *Pursh	IPK	LIN 1550	USA	2n = 18 [17]	
16	*L. austriacum *L.	IPK	LIN 1608	Germany	2n = 18 [17]	
17	*L. austriacum *L.	Wild population, collected by A. A. Svetlova		Russian Federation, Rostov	2n = 18 (present study)	
18	*L. austriacum *L. subsp.* euxinum* (Juz.) Ockendon	IPK	LIN 1546	Ukraine	2n = 18 [17]	
19	*L. austriacum *L.	Wild population, collected by A. A. Svetlova		Crimea	2n = 18 (present study)	
20	*L. austriacum* L.	Wild population, collected by A. A. Svetlova		Ukraine	2n = 18 (present study)	
21	*L. amurense* Alef.	BGI	outdoors cultivated collection	Russian Federation, Far East, near Pokrovka settlement	2n= 18 [17]	

Sect. *Dasylinum *						
22	*L*. *hirsutum *L. subsp.* hirsutum *L.	IPK	LIN 1676	Hungary	2n = 16 [35]	
23	*L*. *hirsutum *subsp.* hirsutum* L.	IPK	LIN1649	Romania	2n = 16 [36]	
24	*L. hirsutum *L. subsp.* pseudoanatolicum* P.H.Davis	Wild population, collected by M. Pavelka		Turkey, Karaman	2n = 32 (present study)	
25	*L. hirsutum *L. subsp.* anatolicum* (Boiss.) Hayek	Wild population, collected by M. Pavelka		Turkey, Aksaray	2n = 32 (present study)	

Sect. *Linum *						
26	*L. marginale *A.Cunn. ex Planch	IPK	LIN 1920	Australia		2n = 84 (present study)
27	*L. narbonense *L.	IPK	LIN 2002	Unknown		2n = 28 [14]
28	*L. narbonense* L.	IPK	LIN1653	France		2n = 28 [14]
29	*L. decumbens *Desf.	IPK	LIN 1754	Italy		2n = 16 [14]
30	*L. decumbens *Desf.	IPK	LIN1913	Italy		2n = 16 [14]
31	*L. grandiflorum* Desf.	IPK	LIN 2000	Unknown		2n = 16 [14]
32	*L. grandiflorum* Desf.	IPK	LIN 974	Unknown		2n = 16 [14]
33	*L. angustifolium* Huds.	IPK	LIN 1692	France		2n = 30 (present study)
34	*L. angustifolium* Huds.	VNIIL	K 5695	Unknown		2n = 30 (present study)
35	*L. angustifolium *Huds.	VNIIL	K 3108	Belgium		2n = 30 (present study)
36	*L. angustifolium *Huds	IGC	15	Belarus		2n = 30 [14]
37	*L. angustifolium *Huds.	VNIIL	K 4731	East Germany		2n = 30 (present study)
38	*L. bienne *Mill. (syn.* L. angustifolium* Huds.)	IGC	14	Hungary		2n = 30 [14]
39	*L. usitatissimum *L. subsp.* bienne* Mill. Snankev.	VIR	u-303794	Unknown	winter flax	2n = 30 (present study)
40	*L. usitatissimum convar.crepitans* [Boenningh.] Kulpa et Danert	IGC	260	Belarus	dehiscent flax	2n = 30 (present study)
41	*L. usitatissimum convar.crepitans* [Boenningh.] Kulpa et Danert	IPK	LIN 119	Portugal	dehiscent flax	2n = 30 (present study)
42	*L. usitatissimum* L. variety Giza	IPK	LIN 277	Egypt	large seeded flax	2n = 30 (present study)
43	*L. usitatissimum* L. G. 12 Ruzokvety	IPK	LIN 633	Czechoslovakia	dual-purpose flax	2n = 30 (present study)
44	*L. usitatissimum* L.,variety Colchidsky 1	VIR	u 099845.	Abkhazia	winter flax	2n = 30 (present study)
45	*L. usitatissimum *L.	VNIIL	k 7131	Morocco	large seeded flax	2n = 30 (present study)

Sect. *Stellerolinum *						
46	*L. stelleroides *Planchon	BGI	Outdoors cultivated collection	Russian Federation, Far East, Telyakovsky Inlet	2n = 20 (present study)	
47	*L. stelleroides* Planchon	BGI	Outdoors cultivated collection	Russian Federation, Far East, near Kraskino settlement	2n = 20 (present study)	
