# Supplementary material for: Retrotransposon-Based Molecular Markers for Analysis of Genetic Diversity within the Genus Linum
Source: Biomed Res Int. 2014 Aug 27;2014:231589. doi: 10.1155/2014/231589 (PMC4163409; doi:10.1155/2014/231589)
Supplement: Supplementary file 1 — Sequences of SSAP PCR products. [file 231589.f1.docx]

**Supplementary materials.** Sequences of SSAP PCR products.

Alignments were performed using NCBI Nucleotide BLAST (http://blast.ncbi.nlm.nih.gov/Blast.cgi).

Yellow – sequences with a significantly similarity to the sequences of *Cassandra*, *FL1a* and *FL1b* retrotransposons.

Adapter sequence:

TCGTGGGATCTATACTTTCGGGTTGAGAATCGAGTAAAC

*Sample 1: TMR 1919 - 1*

ccaaaataaatctgtgagggattagttatgcccaaaccggacaatatcttgtcggaaaagtacggggtgttacaagtggtatccgagcctctctgcatccctcttgaacgatggtggggctaaccgcaatgaggacgttgagtcctatggggggtgtaatgtaacacgttaggcaaatcccacattgacaaagcgcaagagagacttatgggctagaaggaacaatccgtccctaactaacaagacgcatttTCGTGGGATCTATACTTTCGGGTTGAGAATCGAGTAAAC

Linum usitatissimum retrotransposon Cassandra, complete sequence

Sequence ID: [gb|DQ767972.1|](http://www.ncbi.nlm.nih.gov/nucleotide/110559194?report=genbank&log$=nuclalign&blast_rank=1&RID=WHZ68EFE11N)Length: 632Number of Matches: 23

Query 1 CCAAAATAAATCTGTGAGGGATTAGTTATGCCCAAACCGGACAATATCTTGTCGGAAAAG 60

||||||||||||||||||||||||||||||||||||||||||||||||||||||||||||

Sbjct 203 CCAAAATAAATCTGTGAGGGATTAGTTATGCCCAAACCGGACAATATCTTGTCGGAAAAG 262

Query 61 TACGGGGTGTTACAAGTGGTATCCGAGCCTCTCTGCATCCCTCTTGAACGATGGTGGGGC 120

||||||||||||||||||||||||||||||||||||||||||||||||||||||||||||

Sbjct 263 TACGGGGTGTTACAAGTGGTATCCGAGCCTCTCTGCATCCCTCTTGAACGATGGTGGGGC 322

Query 121 TAACCGCAATGAGGACGTTGAGTCCTATGGGGGGTGTAATGTAACACGTTAGGCAAATCC 180

||||||||||||||||||||||||||||||||||||||||||||||||||||||| ||||

Sbjct 323 TAACCGCAATGAGGACGTTGAGTCCTATGGGGGGTGTAATGTAACACGTTAGGCATATCC 382

Query 181 CACATTGACAAAGCGCAAGAGAGACTTATGGGCTAGAAGGAACAATCCGTCCCTAACTAA 240

|| |||||||||| |||||||||||||||||||||||||||||||||||||||||||||

Sbjct 383 CATATTGACAAAGTACAAGAGAGACTTATGGGCTAGAAGGAACAATCCGTCCCTAACTAA 442

Query 241 CAAGACGCATTTTCG 255

|||||||||||||||

Sbjct 443 CAAGACGCATTTTCG 457

*Sample 2: TMR 1919 – 2*

TgcaggggtagttatgcccaaagtggacaatatcttgtcggaaaagtacgggcagttacagtaacaattcatttttaacatatatatgagtctctagtatactaatgttggcaacacggcaggtggttcaaaaatgacatccttacatccaagacatccataaatagaatgagaacactaaacatgcattcggtaaccataaactaaactcaagagggagttttgaaaagtaaggaaaaaaattcaacagtattactcacaagttatgtacccatcaattaagaagcaccctaattcaccacacacttgaacaggacattgtcctcaatggaccaaacaatcaagtgagacagggccagaaaatatggtactaacttggagatcgtgatagtgagcacatgtagagtctgggcttggactaaggctctattcagacggcaagtgagatcgtagagtctgggcttggactaaggctctaTCGTGGGATCTATACTTTCGGGTTGAGAATCGAGTAAAC

Linum usitatissimum retrotransposon Cassandra, complete sequence

Sequence ID: [gb|DQ767972.1|](http://www.ncbi.nlm.nih.gov/nucleotide/110559194?report=genbank&log$=nuclalign&blast_rank=1&RID=WHSA5FAK11N)Length: 632Number of Matches: 29

Query 9 TAGTTATGCCCAAAGTGGACAATATCTTGTCGGAAAAGTACGGG 52

|||||||||||||| ||||||||||||||||||||||||||||

Sbjct 225 TAGTTATGCCCAAACCGGACAATATCTTGTCGGAAAAGTACGGG 268

Query 9 TAGTTATGCCCAAAGTGGACAATATCTTGTCGGAAAAGTACGGG 52

|||||||||||||| ||||||||||||||||||||||||||||

Sbjct 581 TAGTTATGCCCAAACCGGACAATATCTTGTCGGAAAAGTACGGG 624

*Sample 3: TMR 1919 – 3*

CATGGAGGGGCTTCGGGTGCAGTTTGAGGATCTGTATACCGGGTATCGGAGCCAGAGAGAGGGTTGAGGTTGTGTACTATGTTTGATGTACTTTCCTTTTTTCGTGGGATCTATACTTTCGGGTTGAGAATCAANTAAAA

*Sample 4: Stormont cirrus - 1*

agttaatcgtgctcggatgggagcagtgcagaaatgggtgacctcctaggaattcattgttctgtatccgaaataaatccgtgcaggggtagttatgcccaaagtggacaatatcttgtcggaaaagtacgggcagttacagtaacaattcatttttaacatatatatgagtctctagtatactaatgttggcaacacggcagg

Linum usitatissimum retrotransposon Cassandra, complete sequence

Sequence ID: [gb|DQ767972.1|](http://www.ncbi.nlm.nih.gov/nucleotide/110559194?report=genbank&log$=nuclalign&blast_rank=1&RID=WHZE1NZV114)Length: 632Number of Matches: 20

Query 2 GTTAATCGTGCTCGGATGGGAGCAGTGCAGAAATGGGTGACCTCCTAGGAATTCATTGTT 61

|||||||| |||||| ||||||||||||| ||||||||||||||||||||| ||| | ||

Sbjct 137 GTTAATCGCGCTCGGGTGGGAGCAGTGCATAAATGGGTGACCTCCTAGGAAGTCACTATT 196

Query 62 CTGTATCCGAAATAAATCCGTGCAGGGGTAGTTATGCCCAAAGTGGACAATATCTTGTCG 121

||| |||| ||||||||| ||| || |||||||||||||| ||||||||||||||||

Sbjct 197 CTGCATCCAAAATAAATCTGTGAGGGATTAGTTATGCCCAAACCGGACAATATCTTGTCG 256

Query 122 GAAAAGTACGGG 133

||||||||||||

Sbjct 257 GAAAAGTACGGG 268

Query 2 GTTAATCGTGCTCGGATGGGAGCAGTGCAGAAATGGGTGACCTCCTAGGAATTCATTGTT 61

|||||||| |||||| ||||||||||||| ||||||||||||||||||||| ||| | ||

Sbjct 493 GTTAATCGCGCTCGGGTGGGAGCAGTGCATAAATGGGTGACCTCCTAGGAAGTCACTATT 552

Query 62 CTGTATCCGAAATAAATCCGTGCAGGGGTAGTTATGCCCAAAGTGGACAATATCTTGTCG 121

||| |||| ||||||||| ||| || |||||||||||||| ||||||||||||||||

Sbjct 553 CTGCATCCAAAATAAATCTGTGAGGGATTAGTTATGCCCAAACCGGACAATATCTTGTCG 612

Query 122 GAAAAGTACGGG 133

||||||||||||

Sbjct 613 GAAAAGTACGGG 624

*Sample 5: Stormont cirrus - 2*

aaaaaaaatccgtgagggattaactatgcccaaagcgaaaaatatcttgtcggaaaaggtcggggtgttacaagtggtatcagagcctggtctccctcaatctcgtcaggaaattgacgagagactaggagtcggtcatgttttaagaagtggaaagtgttttgaaaaggtttatgtattttagcaagtctcacgtccactccaccgaatcagctacttctgcaagaaggtactcatagttcaatgtcTTCGTGGGATCTATACTTTCGGGTTGAGAATCGAGTAAAC

Linum usitatissimum retrotransposon Cassandra, complete sequence

Sequence ID: [gb|DQ767972.1|](http://www.ncbi.nlm.nih.gov/nucleotide/110559194?report=genbank&log$=nuclalign&blast_rank=1&RID=WHZPBC85114)Length: 632Number of Matches: 22

Query 1 AAAAAAAATCCGTGAGGGATTAACTATGCCCAAAGCGAAAAATATCTTGTCGGAAAAGGT 60

|||| ||||| ||||||||||| |||||||||| || | ||||||||||||||||||

Sbjct 205 AAAATAAATCTGTGAGGGATTAGTTATGCCCAAACCGGACAATATCTTGTCGGAAAAGTA 264

Query 61 CGGGGTGTTACAAGTGGTATCAGAGCCT 88

||||||||||||||||||||| ||||||

Sbjct 265 CGGGGTGTTACAAGTGGTATCCGAGCCT 292

Query 1 AAAAAAAATCCGTGAGGGATTAACTATGCCCAAAGCGAAAAATATCTTGTCGGAAAAGGT 60

|||| ||||| ||||||||||| |||||||||| || | ||||||||||||||||||

Sbjct 561 AAAATAAATCTGTGAGGGATTAGTTATGCCCAAACCGGACAATATCTTGTCGGAAAAGTA 620

Query 61 CGGGGTGTTACA 72

||||||||||||

Sbjct 621 CGGGGTGTTACA 632

*Sample 6: Stormont cirrus - 3*

aaataaatctgtgagggattagttatgcctaaagcagacaatatcttgtcacaaaaggacggggtgttacacacagtatgttaggaggtcggggtgttacagatagtgtcttagaattcgaTCGTGGGATCTATACTTTCGGGTTGAGAATCGAGTAAA

Linum usitatissimum retrotransposon Cassandra, complete sequence

Sequence ID: [gb|DQ767972.1|](http://www.ncbi.nlm.nih.gov/nucleotide/110559194?report=genbank&log$=nuclalign&blast_rank=1&RID=WHZS1W0P114)Length: 632Number of Matches: 19

Query 1 AAATAAATCTGTGAGGGATTAGTTATGCCTAAAGCAGACAATATCTTGTCACAAAAGGAC 60

||||||||||||||||||||||||||||| ||| | |||||||||||||| ||||| ||

Sbjct 206 AAATAAATCTGTGAGGGATTAGTTATGCCCAAACCGGACAATATCTTGTCGGAAAAGTAC 265

Query 61 GGGGTGTTACA 71

|||||||||||

Sbjct 266 GGGGTGTTACA 276

Query 1 AAATAAATCTGTGAGGGATTAGTTATGCCTAAAGCAGACAATATCTTGTCACAAAAGGAC 60

||||||||||||||||||||||||||||| ||| | |||||||||||||| ||||| ||

Sbjct 562 AAATAAATCTGTGAGGGATTAGTTATGCCCAAACCGGACAATATCTTGTCGGAAAAGTAC 621

Query 61 GGGGTGTTACA 71

|||||||||||

Sbjct 622 GGGGTGTTACA 632

*Sample 7: L. angustifolium* K 5695 - 1

GCCTTCGGGNGCAGCTTTGAGGNATCTGTTATACCGGGTATCGGAGCCAGAGAGAGGGTTGAGGTTGTGTACTATGTTTGATGTACTTTCCTTTTTTCGTGGGATCTATACTTTCGGGTTGAGAATCGAGTAAAC

*Sample 8: L. angustifolium* K 5695 - 2

TgcaggggtagttatgcccaaagtggacaatatcttgtcggaaaagtacgggcagttacagtaacaattcatttttaacatatatatgagtctctagtatactaatgttggcaacacggcaggtggttcaaaaatgacatccttacatccaagacatccataaatagaatgagaacactaaacatgcattcggtaaccataaactaaactcaagagggagttttgaaaagtaaggaaaaaaattcaacagtattactcacaagttatgtacccatcaattaagaagcaccctaattcaccacacacttgaacaggacattgtcctcaatggaccaaacaatcaagtgagacagggccagaaaatatggtactaacttggagatcgtgatagtgagcacatgtagagtctgggcttggactaaggctctattcagacggcaagtgagatcgtagagtctgggcttggactaaggctctaTCGTGGGATCTATACTTTCGGGTTGAGAATCGAGTAAAC

Linum usitatissimum retrotransposon Cassandra, complete sequence

Sequence ID: [gb|DQ767972.1|](http://www.ncbi.nlm.nih.gov/nucleotide/110559194?report=genbank&log$=nuclalign&blast_rank=1&RID=WJ03FYYP114)Length: 632Number of Matches: 29

Query 9 TAGTTATGCCCAAAGTGGACAATATCTTGTCGGAAAAGTACGGG 52

|||||||||||||| ||||||||||||||||||||||||||||

Sbjct 225 TAGTTATGCCCAAACCGGACAATATCTTGTCGGAAAAGTACGGG 268

Query 9 TAGTTATGCCCAAAGTGGACAATATCTTGTCGGAAAAGTACGGG 52

|||||||||||||| ||||||||||||||||||||||||||||

Sbjct 581 TAGTTATGCCCAAACCGGACAATATCTTGTCGGAAAAGTACGGG 624

*Sample 9: L. bienne 14 - 1*

GGGCCTTCGGGGTGCAGTTTGAGGATCTGTATACCGGGTATCGGAGCCAGAGAGAGGGTTGAGGTTGTGTACTATGTTTGATGTACTTTCCTTTTTTCGTGGGATCTATACTTTCGGGTTGAGAATCGAGTAAAC

*Sample 10: L. bienne 14 - 2*

ggattagttatgcccaaaccggacaattatcttgtcggaaaagtacggggtgttacaagtggtatccgagcctctctgcatccctcttgaacgatggtggggctaaccgcaatgaggacgttgagtcgtatggggggtgtaatgtaacacgttaggcaaatcccacattgacaaagcgcaagagagacttatgggctagaaggaacaatccgtccctaactaacaagacgcatttTCGTGGGATCTATACTTTCGGGTTGAGAATCGAGTAAAC

Linum usitatissimum retrotransposon Cassandra, complete sequence

Sequence ID: [gb|DQ767972.1|](http://www.ncbi.nlm.nih.gov/nucleotide/110559194?report=genbank&log$=nuclalign&blast_rank=1&RID=WJ08BAFK11N)Length: 632Number of Matches: 20

Query 1 GGATTAGTTATGCCCAAACCGGACAATTATCTTGTCGGAAAAGTACGGGGTGTTACAAGT 60

||||||||||||||||||||||||||| ||||||||||||||||||||||||||||||||

Sbjct 221 GGATTAGTTATGCCCAAACCGGACAAT-ATCTTGTCGGAAAAGTACGGGGTGTTACAAGT 279

Query 61 GGTATCCGAGCCTCTCTGCATCCCTCTTGAACGATGGTGGGGCTAACCGCAATGAGGACG 120

||||||||||||||||||||||||||||||||||||||||||||||||||||||||||||

Sbjct 280 GGTATCCGAGCCTCTCTGCATCCCTCTTGAACGATGGTGGGGCTAACCGCAATGAGGACG 339

Query 121 TTGAGTCGTATGGGGGGTGTAATGTAACACGTTAGGCAAATCCCACATTGACAAAGCGCA 180

||||||| |||||||||||||||||||||||||||||| |||||| |||||||||| ||

Sbjct 340 TTGAGTCCTATGGGGGGTGTAATGTAACACGTTAGGCATATCCCATATTGACAAAGTACA 399

Query 181 AGAGAGACTTATGGGCTAGAAGGAACAATCCGTCCCTAACTAACAAGACGCATTTTCG 238

||||||||||||||||||||||||||||||||||||||||||||||||||||||||||

Sbjct 400 AGAGAGACTTATGGGCTAGAAGGAACAATCCGTCCCTAACTAACAAGACGCATTTTCG 457

Query 138 TGTAATGTAACACGTTAGGCAAATCCCACATTGACAAAGCGCAAGAGAGACTTATGGGCT 197

||||||||||||||||||||| |||||| |||||||||| |||||||||||||||||||

Sbjct 1 TGTAATGTAACACGTTAGGCATATCCCATATTGACAAAGTACAAGAGAGACTTATGGGCT 60

Query 198 AGAAGGAACAATCCGTCCCTAACTAACAAGACGCATTTTCG 238

|||||||||||||||||||||||||||||||||||||||||

Sbjct 61 AGAAGGAACAATCCGTCCCTAACTAACAAGACGCATTTTCG 101

Query 1 GGATTAGTTATGCCCAAACCGGACAATTATCTTGTCGGAAAAGTACGGGGTGTTACA 57

||||||||||||||||||||||||||| |||||||||||||||||||||||||||||

Sbjct 577 GGATTAGTTATGCCCAAACCGGACAAT-ATCTTGTCGGAAAAGTACGGGGTGTTACA 632
